# Thinking outside the curve, part II: modeling fetal-infant mortality

**DOI:** 10.1186/1471-2393-10-44

**Published:** 2010-08-12

**Authors:** Richard Charnigo, Lorie W Chesnut, Tony LoBianco, Russell S Kirby

**Affiliations:** 1 Departments of Statistics and Biostatistics, University of Kentucky Lexington, KY 40506-0027, USA; 2 Department of Epidemiology, University of Kentucky Lexington, KY 40536-0003, USA; 3 Interdisciplinary Human Development Institute, University of Kentucky Lexington, KY 40506-0051, USA; 4 Department of Community and Family Health, University of South Florida Tampa, FL 33612, USA

## Abstract

**Background:**

Greater epidemiologic understanding of the relationships among fetal-infant mortality and its prognostic factors, including birthweight, could have vast public health implications. A key step toward that understanding is a realistic and tractable framework for analyzing birthweight distributions and fetal-infant mortality. The present paper is the second of a two-part series that introduces such a framework.

**Methods:**

We propose estimating birthweight-specific mortality within each component of a normal mixture model representing a birthweight distribution, the number of components having been determined from the data rather than fixed *a priori*.

**Results:**

We address a number of methodological issues related to our proposal, including the construction of confidence intervals for mortality risk at any given birthweight within a component, for odds ratios comparing mortality within two different components from the same population, and for odds ratios comparing mortality within analogous components from two different populations. As an illustration we find that, for a population of white singleton infants, the odds of mortality at 3000 g are an estimated 4.15 times as large in component 2 of a 4-component normal mixture model as in component 4 (95% confidence interval, 2.04 to 8.43). We also outline an extension of our framework through which covariates could be probabilistically related to mixture components. This extension might allow the assertion of approximate correspondences between mixture components and identifiable subpopulations.

**Conclusions:**

The framework developed in this paper does not require infants from compromised pregnancies to share a common birthweight-specific mortality curve, much less assume the existence of an interval of birthweights over which all infants have the same curve. Hence, the present framework can reveal heterogeneity in mortality that is undetectable via a contaminated normal model or a 2-component normal mixture model.

## Background

A recent report shows a slight decline in the rate of infants with low birthweights (less than 2500 g) in the United States, with a rate of 8.2 percent in 2007 compared to 8.3 percent in 2006 [1]. While the rate for extremely low (ELBW; <1000 g) and very low birthweights (VLBW; 1000-1500 g) was unchanged at 1.5 percent, the rate for moderately low birthweights (MLBW; 1500-2500 g) declined from 6.8 to 6.7 percent [1]. Data on the proportions of normal (NBW; 2500-4000 g) and high birthweights (HBW; >4000 g) were not provided. If confirmed in the final vital records data, the decline in the low birthweight rate will be the first in many years. National Center for Health Statistics (NCHS) records indicate that low birthweight rates have been rising since 1984, when the rate was 6.7 percent [1].

Perinatal epidemiologists have long recognized birthweight as one of several factors related to fetal growth, and ultimately, infant survival and development [2-4]. However, categories such as ELBW and VLBW, while useful for descriptive purposes, are not completely satisfactory for representing the birthweight distribution of a population, much less assessing the relationship between birthweight and fetal-infant mortality. First, cutoffs such as 1500 g and 2500 g are arbitrary and introduce an artificial discreteness to a naturally continuous phenomenon: presumably fetal-infant mortality risk decreases only incrementally as one moves from, for example, 2499 g to 2501 g. Second, there may still be heterogeneity at any fixed birthweight: some infants born at, say, 2499 g may be at higher risk than other infants born at 2499 g.

The preceding considerations motivate a new framework for modeling birthweight distributions and fetal-infant mortality. This is the second paper in a two-part series that introduces such a framework. In the first paper, we proposed a normal mixture model for birthweight distribution:

(1) $$\sum_{j=1}^{k}p_{j}f(x;\mu_{j},\sigma_{j}),$$

where *k *is the number of components, *x *is birthweight, *p*_*j *_is the fraction of births in component *j*, *μ*_*j *_is the mean of the birthweights in component *j*, *σ*_*j *_is the standard deviation of the birthweights in component *j*, and *f *(*x*; *μ*_*j*_, *σ*_*j*_) is the probability density for a normal distribution with mean *μ*_*j *_and standard deviation *σ*_*j*_. What distinguished our proposal from the contaminated normal model of Umbach and Wilcox [5] and the 2-component normal mixture model of Gage and Therriault [6] was that the number of components was not fixed *a priori *but rather determined from the data using the Flexible Information Criterion (FLIC) (Pilla and Charnigo, Consistent estimation and model selection in semiparametric mixtures, submitted). We also showed how to construct confidence intervals for *p*_*j*_, *μ*_*j*_, and *σ*_*j *_(1 <= *j *<= *k*) based on multiple samples from the same population, even if those samples overlapped.

Here we consider estimating birthweight-specific mortality curves within each component of the normal mixture model in Equation (1). We begin by generalizing Gage's parametric mixtures of logistic regressions (PMLR) technique [7] to accommodate a normal mixture model with more than two components. We proceed to show how confidence bounds can be constructed for birthweight-specific mortality curves. We then provide formulas for estimating mortality odds ratios comparing populations on the same component, such as

odds of mortality at 2500 g in component 3 (white heavy smoking population) divided by

odds of mortality at 2500 g in component 3 (white general population),

or comparing components in the same population, such as

odds of mortality at 1000 g in component 2 (white heavy smoking population) divided by

odds of mortality at 1000 g in component 1 (white heavy smoking population).

Being able to estimate the latter kind of odds ratio - in other words, being able to assert that some infants in a population are at higher risk than others, even when they are of the same birthweight - is the main advantage of modeling a birthweight distribution as we have proposed, rather than using a contaminated normal model [5] or a 2-component normal mixture model [6]. Thus, our two-part series provides a modeling framework through which heterogeneity in mortality can be revealed that might otherwise remain undetected.

## Results

### 1. Mortality risk estimation from a single sample

#### a. Description of the methodology

Gage developed a parametric mixtures of logistic regressions (PMLR) technique to estimate mortality risk as a function of birthweight within each of two components in a normal mixture model describing a birthweight distribution [7]. Although PMLR was formulated for a 2-component model, we generalize it to *k *components as follows.

The risk function or birthweight-specific mortality curve for component *j *(1 <= *j *<= *k *) is

(2) $$r_{j}(x)={\text{logit}}^{-1}[p_{j}(x)]=\exp[p_{j}(x)]/(1+\exp[p_{j}(x)]),$$

where *x *represents birthweight and *p*_*j*_(*x*) is a polynomial whose coefficients must be estimated. By the law of total probability [8], the risk function for the population overall is

(3) $$\sum_{j=1}^{k}r_{j}(x)p_{j}f(x;\mu_{j},\sigma_{j})/\sum_{j=1}^{k}p_{j}f(x;\mu_{j},\sigma_{j}).$$

Gage took *p*_*j*_(*x*) to be a second-degree polynomial, allowing the birthweight-specific mortality curves for each of his two components to be U-shaped [7]. However, since our framework permits more than two components, we are reluctant to assume that a U-shaped pattern should prevail within every component. Thus, we take *p*_*j*_(*x*) to be a fourth-degree polynomial, which accommodates up to two changes in convexity for each birthweight-specific mortality curve.

Since estimates of *p*_*j*_, *μ*_*j*_, and *σ*_*j *_(1 <= *j *<= *k*) are required to calculate the Flexible Information Criterion (FLIC) (Pilla and Charnigo, Consistent estimation and model selection in semiparametric mixtures, submitted) when determining the number of components, we may assume that these estimates are now available. We then employ the optimization (optim) procedure in version 2.3.1 of R (R Foundation for Statistical Computing, Vienna, Austria, 2006) to estimate *r*_1_(*x*) through *r*_*k*_(*x*) by maximum likelihood conditional on the estimates of *p*_*j*_, *μ*_*j*_, and *σ*_*j *_(1 <= *j *<= *k*). Thus, PMLR represents the second half of a two-stage procedure for modeling birthweight distribution and fetal-infant mortality. Our R code is available upon written request to the corresponding author. Section I of [Additional file 1] provides details on initial value specification for PMLR.

#### b. An illustrative example

We continue the example from Section 2a of Results from the previous paper, involving a data set of size 50,000 from the NCHS Public-Use Perinatal Mortality Data. This data set was a random sample from the population of 202,849 white singletons who were born (or experienced fetal death) from 2000 to 2002 and whose mothers smoked heavily (at least twenty cigarettes per day). Equation (5) in our previous paper shows the estimates of *p*_*j*_, *μ*_*j*_, and *σ*_*j *_(1 <= *j *<= 4) from the FLIC-selected 4-component model. Using these estimates, we employed PMLR as described above to estimate *r*_1_(*x*) through *r*_4_(*x*).

**Figure 1:**
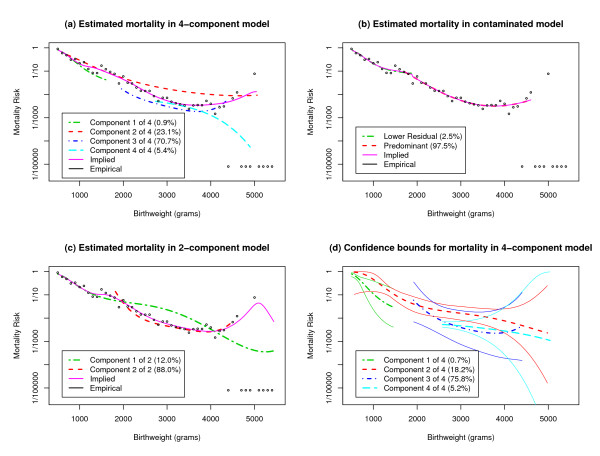
**Mortality for White Singleton Infants with Heavily Smoking Mothers**. (a) Estimated birthweight-specific mortality curves are presented for each component of a 4-component normal mixture model, along with model-implied mortality (a superposition of the estimated birthweight-specific mortality curves) and empirical mortality (crude rates in 100 g bins). The results are based on a single sample of size 50,000 from the population of white singletons born to heavily smoking mothers. (b) and (c) Corresponding results are displayed for a contaminated normal model and a 2-component normal mixture model. (d) Estimated birthweight specific-mortality curves are presented for each component of a 4-component normal mixture model, along with confidence bounds determined by Equations (6) and (7) with *C*_0 _= 4.0 and *φ *= .2465 based on 25 samples of size 50,000.

Figure 1a shows the results. The vertical axis is logarithmic. Estimated birthweight-specific mortality curves for the 4 components are in dashes. We suppress the portions of each curve for birthweights more than three component standard deviations away from the component mean. The model-implied mortality curve, a weighted average of estimated birthweight-specific mortality curves for the four components, is in solid. This curve estimates the population risk function, whose form under the 4-component model is given by Equation (3) with *k *= 4. Empirical mortality, defined here to consist of the crude mortality rates in 100 g bins, is depicted with circles. Crude rates of zero due to extremely small denominators are displayed near the bottom of Figure 1a.

Birthweight-specific mortality appears roughly U-shaped within component 3. The patterns for the other components are decreasing rather than U-shaped, although the decrease for component 2 plateaus in the HBW range. The decrease for component 4 actually becomes steeper in the HBW range, but this seems to be an artifact: the proportion of births in component 4 is small, and there are rather few deaths at large birthweights, so estimating birthweight-specific mortality within component 4 at large birthweights is difficult. The model-implied mortality curve tracks empirical mortality very closely when the denominators for the crude rates are not too small.

#### c. Results from competing models for birthweight distribution

We used the same data set to estimate birthweight-specific mortality curves for the lower residual and predominant distributions in a contaminated normal model (Figure 1b) [5]. Since the estimated proportion of births in the upper residual distribution was less than 1 in 8700, we did not attempt to estimate a birthweight-specific mortality curve for the upper residual distribution.

The model-implied mortality curve generally appears reasonable, although there is an artifact at the threshold of 1700 g, where the lower residual distribution terminates. The contaminated normal model asserts that all infants at any fixed birthweight greater than 1700 g (and less than 5300 g, if one considers the upper residual distribution) have the same mortality risk. Moreover, since the predominant distribution is virtually nonexistent in the VLBW and ELBW ranges, the contaminated normal model cannot detect heterogeneity in mortality risk at any fixed birthweight in the VLBW and ELBW ranges.

We also estimated birthweight-specific mortality curves for the primary and secondary distributions in a 2-component normal mixture (Figure 1c) [6]. The model-implied mortality curve appears reasonable except for the pronounced downturn at 5100 g, which is an artifact of the extremely small denominators above 5000 g.

The 2-component normal mixture can detect heterogeneity in the NBW range and parts of the MLBW and HBW ranges. However, since the primary distribution is virtually nonexistent in the VLBW and ELBW ranges, the 2-component normal mixture cannot detect heterogeneity in those ranges.

### 2. Mortality risk estimation from multiple samples

#### a. Confidence bounds

To quantify uncertainty in the estimation of birthweight-specific mortality, we proceed as follows. First, we draw *N*_*rep *_samples from the population of interest, where each sample consists of birthweight/mortality outcome pairs. Second, we fit a *k*-component normal mixture model to the birthweight data in each sample. Third, we apply PMLR to the birthweight and mortality outcome data in each sample, which yields estimated birthweight-specific mortality curves for that sample. Fourth, we use the *N*_*rep *_sets of estimated birthweight-specific mortality curves to create overall estimates of the risk functions and accompanying confidence bounds, as described below.

Let ${\overset{\wedge }{r}}_{j;1}(x),{\overset{\wedge }{r}}_{j;2}(x),...,{\overset{\wedge }{r}}_{j;N_{rep}}(x)$ denote the estimated birthweight-specific mortality curves for component *j *(1 <= *j *<= *k *) originating from the *N*_*rep *_samples. An overall estimate of the risk function for component *j *is

(4) $${\overset{\wedge }{r}}_{j}(x)={\text{logit}}^{-1}[N_{rep}^{-1}\sum_{s=1}^{N_{rep}}\text{logit}\{{\overset{\wedge }{r}}_{j;s}(x)\}].$$

The rationale for using the logit transformation in Equation (4), as well as in the elements entering Equations (5) and (6) below, is described in Section II of [Additional file 1].

Fixing *x *= *x*_0_, we set *θ *= logit{ *r*_*j*_(*x*_0_)} and define ${\overset{\wedge }{\theta }}_{1},{\overset{\wedge }{\theta }}_{2},...,{\overset{\wedge }{\theta }}_{N_{rep}}$ as logit{${\overset{\wedge }{r}}_{j;1}(x_{0})$}, logit{${\overset{\wedge }{r}}_{j;2}(x_{0})$},...,logit{${\overset{\wedge }{r}}_{j;N_{rep}}(x_{0})$}. With $\overset{\wedge }{\theta }$ and ${\overset{\wedge }{S}}_{\theta }$ denoting the "meta-sample" mean and standard deviation of ${\overset{\wedge }{\theta }}_{1},{\overset{\wedge }{\theta }}_{2},...,{\overset{\wedge }{\theta }}_{N_{rep}}$, we construct a confidence interval for *θ *via either

(5) $$\overset{\wedge }{\theta }+\text{or}-C{\overset{\wedge }{S}}_{\theta }/\sqrt{N_{rep}}$$

or, preferably,

(6) $$\overset{\wedge }{\theta }+\text{or}-\{{\overset{\wedge }{B}}_{\theta }+C{\overset{\wedge }{S}}_{\theta }/\sqrt{N_{rep}}\},$$

where ${\overset{\wedge }{B}}_{\theta }$ is a bias adjustment and *C *is a constant chosen so that the confidence interval has the desired coverage probability (typically 95%).

If Equation (6) is used, we obtain ${\overset{\wedge }{B}}_{\theta }$ by simulation. More specifically, we randomly generate birthweights from $\sum_{j=1}^{k}{\overset{\wedge }{p}}_{j}f(x;{\overset{\wedge }{\mu }}_{j},{\overset{\wedge }{\sigma }}_{j})$, where ${\overset{\wedge }{p}}_{1},{\overset{\wedge }{\mu }}_{1},{\overset{\wedge }{\sigma }}_{1},...,{\overset{\wedge }{p}}_{k},{\overset{\wedge }{\mu }}_{k},{\overset{\wedge }{\sigma }}_{k}$ are the overall estimates of their respective parameters; see Section 2c of Results from the previous paper. Then we use ${\overset{\wedge }{r}}_{1}(x),{\overset{\wedge }{r}}_{2}(x),...,{\overset{\wedge }{r}}_{k}(x)$ from Equation (4) to randomly generate corresponding mortality outcomes. This yields a simulated data set consisting of birthweight/mortality outcome pairs. Fitting a *k*-component normal mixture model to the simulated birthweight data and then applying PMLR to the simulated birthweight and mortality outcome data, we obtain an "estimate" of $\overset{\wedge }{\theta }$, which we call ${\overset{\wedge }{\overset{\wedge }{\theta }}}_{sim}$. We create four more simulated data sets in the same manner, recover the value of ${\overset{\wedge }{\overset{\wedge }{\theta }}}_{sim}$ for each, and then define ${\overset{\wedge }{B}}_{\theta }$ as the average value of $\left|{\overset{\wedge }{\overset{\wedge }{\theta }}}_{sim}-\overset{\wedge }{\theta }\right|$ over the five simulated data sets.

A confidence interval for *r*_*j*_(*x*_0_) is obtained by applying the inverse logit transformation to the confidence interval for *θ*. The above computations can be performed simultaneously at a series of birthweights. Connecting the resulting series of upper confidence limits produces an upper confidence bound for the risk function in component *j*, while connecting the resulting series of lower confidence limits produces a lower confidence bound.

As when constructing confidence intervals for *p*_*j*_, *μ*_*j*_, and *σ*_*j *_(1 <= *j *<= *k*), we can accommodate overlap in the *N*_*rep *_samples by choosing the value of *C *in Equation (6) according to the fraction of the underlying population that each of the *N*_*rep *_samples constitutes. Let *C*_0 _denote the value of *C *that would be chosen if this fraction were negligibly small, and let *C*_*φ *_denote the value that would be chosen if this fraction were equal to *φ*, a positive number less than 1. In the previous paper, we established the relationship

(7) $$C_{\varphi }=C_{0}\sqrt{\varphi N_{rep}/\{1-{\left(1-\varphi \right)}^{N_{rep}}\}}.$$

#### b. Illustrative example

We continue the example from Section 2c of Results from the previous paper, involving *N*_*rep *_= 25 data sets of size 50,000 from the NCHS Public-Use Perinatal Mortality Data. These data sets were random samples from the aforementioned population of 202,849 white singletons whose mothers smoked heavily.

Figure 1d displays overall estimates and confidence bounds for the birthweight-specific mortality curves in a 4-component model for the birthweights of white singletons born to heavily-smoking mothers. We took *C*_0 _= 4.0 (see Section 3a of Results) and *φ *= .2465 = 50,000/202,849. Table 1 presents numerical results at selected birthweights; the odds ratios are estimated as described in Section 2c of Results.

**Table 1:** Mortality for White Singleton Infants with Heavily Smoking Mothers

Quantity	@ 1000 g	@ 2000 g	@ 3000 g	@ 4000 g
Risk in component 1: logit^-1^{$\overset{\wedge }{\theta }$} [point estimate] Confidence interval	110.1 (23.2, 392.2)	---	---	---

Risk in component 2: logit^-1^{$\overset{\wedge }{\theta }$} [point estimate] Confidence interval	460.3 (138.3, 819.2)	35.9 (16.9, 74.7)	16.2 (8.2, 31.5)	7.6 (2.3, 25.0)

Risk in component 3: logit^-1^{$\overset{\wedge }{\theta }$} [point estimate] Confidence interval	---	41.3 (6.1, 232.1)	4.0 (0.7, 20.8)	2.4 (0.2, 29.6)

Risk in component 4: logit^-1^{$\overset{\wedge }{\theta }$} [point estimate] Confidence interval	---	---	4.7 (3.0, 7.2)	2.8 (0.3, 28.3)

Odds ratio, component 1 vs. component 2: exp{$\overset{\wedge }{\theta }$} [point estimate] Confidence interval	0.15 (0.01, 2.46)	---	---	---

Odds ratio, component 2 vs. component 3: exp{$\overset{\wedge }{\theta }$} [point estimate] Confidence interval	---	0.87 (0.06, 12.7)	4.13 (0.41, 42.0)	3.19 (0.09, 115)

Odds ratio, component 2 vs. component 4: exp{$\overset{\wedge }{\theta }$} [point estimate] Confidence interval	---	---	3.51 (1.44, 8.56)	2.74 (0.14, 53.1)

Odds ratio, component 3 vs. component 4: exp{$\overset{\wedge }{\theta }$} [point estimate] Confidence interval	---	---	0.85 (0.19, 3.79)	0.86 (0.04, 21.1)

Mortality risks and odds ratios are estimated at selected birthweights, based on 25 samples of size 50,000 from the population of white singletons born to heavily smoking mothers. Confidence intervals are constructed using Equations (6) and (7) with *C*_0 _= 4.0 and *φ *= .2465.

Figure 1d reveals considerable uncertainty in estimating birthweight-specific mortality, especially in the HBW range. However, the confidence bounds for components 2 and 4 have no overlap in the lower part of the NBW range, indicating heterogeneity in mortality risk despite the uncertainty in estimation. That the confidence bounds are so wide is partly due to the large *φ*, which in turn is a consequence of the small population. Section 3b of Results will present another example in which the population is considerably larger and *φ *is much smaller.

#### c. Estimating odds ratios

To estimate an odds ratio comparing components in the same population, such as

odds of mortality at 1000 g in component 2 (white heavy smoking population) divided by

odds of mortality at 1000 g in component 1 (white heavy smoking population),

we apply Equation (6) with the following modifications. Instead of identifying *θ *with logit{ *r*_*j*_(*x*_0_) }, we take *θ *= logit{ *r*_*j*__1_(*x*_0_) } - logit{ *r*_*j*__2_(*x*_0_) }, where 1 <= *j*_1_, *j*_2 _<= *k*. Then exp{*θ*} equals the mortality odds in component *j*_1 _at birthweight *x*_0 _divided by the mortality odds in component *j*_2_. Hence, exp{$\overset{\wedge }{\theta }$} is an estimate of the odds ratio, and

(8) $$\exp\{\overset{\wedge }{\theta }+\text{or}-\{{\overset{\wedge }{B}}_{\theta }+C{\overset{\wedge }{S}}_{\theta }/\sqrt{N_{rep}}\}\}$$

is a confidence interval.

To estimate an odds ratio comparing populations on the same component, such as

odds of mortality at 2500 g in component 3 (white heavy smoking population) divided by

odds of mortality at 2500 g in component 3 (white general population),

we define *θ*_1 _= logit{*r*_*j*_(*x*_0_)} for the first population and *θ*_2 _= logit{*r*_*j*_(*x*_0_)} for the second population. Then exp{*θ*_1 _- *θ*_2_} equals the mortality odds in component *j *of the first population at birthweight *x*_0 _divided by the mortality odds in component *j *of the second population. Hence, $\exp\{{\overset{\wedge }{\theta }}_{1}-{\overset{\wedge }{\theta }}_{2}\}$ is an estimate of the odds ratio, and

(9) $$\exp\{{\overset{\wedge }{\theta }}_{1}-{\overset{\wedge }{\theta }}_{2}+\text{or}-\{{\overset{\wedge }{B}}_{\theta_{1}}+{\overset{\wedge }{B}}_{\theta_{2}}+C\sqrt{{\overset{\wedge }{S}}_{\theta_{1}}^{2}/N_{rep}+{\overset{\wedge }{S}}_{\theta_{2}}^{2}/N_{rep}}\}\}$$

is a confidence interval. Subscripts 1 and 2 in Equation (9) identify the populations to which the "meta-sample" means, standard deviations, and bias adjustments pertain.

### 3. Further illustrations

#### a. Simulation study to calibrate confidence intervals

We simulated 25 overlapping data sets of size 50,000, the degree of overlap consistent with a population of 200,000, based on the specifications in Table 2. The mixture density and the risk functions were chosen to mimic the patterns actually observed for white singletons born to heavily-smoking mothers; see panel b of Figure One from the previous paper and Figure 1d of the present paper. For each of various *C *between 2.0 and 5.0, we used Equation (6) to form confidence intervals for mortality risks at selected birthweights, namely *r*_1_(*μ*_1 _- *σ*_1_), *r*_1_(*μ*_1_), *r*_1_(*μ*_1 _+ *σ*_1_), *r*_2_(*μ*_2 _- *σ*_2_), *r*_2_(*μ*_2_), *r*_2_(*μ*_2 _+ *σ*_2_), *r*_3_(*μ*_3 _- *σ*_3_), *r*_3_(*μ*_3_), *r*_3_(*μ*_3 _+ *σ*_3_), *r*_4_(*μ*_4 _- *σ*_4_), *r*_4_(*μ*_4_), and *r*_4_(*μ*_4 _+ *σ*_4_). Above, *μ*_*j *_and *σ*_*j *_denote the mean and standard deviation of the birthweights in component *j *(1 <= *j *<= 4). This was repeated nine more times, and we tabulated how many of the 120 = 12 × 10 confidence intervals contained their targets. Confidence intervals were also formed using Equation (5) for comparative purposes. The above steps were repeated with overlapping data sets consistent with a population of 1,000,000 and with nonoverlapping data sets consistent with an effectively infinite population.

**Table 2:** Mixture Model and Mortality Functions for Simulation Study

Model feature	Specification for simulation study
Probability density for mixture model	.007 *f*(*x;*832,210) +.182 *f*(*x;*2772,740) +.758 *f*(*x;*3170,417) +.052 *f*(*x;*3804,413)

Risk within component 1	*r*_1_(*x*) = logit ^-1^(-4.6975 -0.2362 *z *+ 0.3994 *z*^2 ^+ 0.1690 *z*^3 ^+ 0.1328 *z*^4^)

Risk within component 2	*r*_2_(*x*) = logit ^-1^(-4.0962 -0.7496 *z *- 0.0289 *z*^2 ^- 0.1094 *z*^3 ^+ 0.0918 *z*^4^)

Risk within component 3	*r*_3_(*x*) = logit ^-1^(-5.7538 -1.7275 *z *+ 1.6269 *z*^2 ^+ 0.1897 *z*^3 ^- 0.0249 *z*^4^)

Risk within component 4	*r*_4_(*x*) = logit ^-1^(-5.3285 -0.2786 *z *- 0.1979 *z*^2 ^+ 0.0535 *z*^3 ^+ 0.0773 *z*^4^)

The probability density for the mixture model used in our simulation study is specified, as are the mortality risk functions associated with the mixture model components. Above, *z *is defined as (*x *- 3000)/1000, where *x *is birthweight in grams.

The results are summarized in Table 3. With an effectively infinite population, only 75.0% of the confidence intervals formed using Equation (5) contained their targets at *C *= 5.0. On the other hand, the confidence intervals formed using Equation (6) contained their targets 95.0% of the time at *C *= 4.0. The latter finding provided the rationale for taking *C*_0 _= 4.0 when constructing confidence intervals for mortality risks in our examples with real data.

**Table 3:** Confidence Interval Coverage Probabilities in Simulation Study

*C*	Population Size	Bias adjustment included	Bias adjustment omitted
		
		Number & Percentage of Intervals Containing Targets (mortality risks)	Number & Percentage of Intervals Containing Targets (mortality risks)
2.0	200,000	69 (57.5)	26 (21.7)
	
	1,000,000	92 (76.7)	26 (21.7)
	
	Infinite	92 (76.7)	43 (35.8)

2.5	200,000	78 (65.0)	29 (24.2)
	
	1,000,000	100 (83.3)	37 (30.8)
	
	Infinite	96 (80.0)	47 (39.2)

3.0	200,000	84 (70.0)	32 (26.7)
	
	1,000,000	106 (88.3)	44 (36.7)
	
	Infinite	102 (85.0)	56 (46.7)

3.5	200,000	89 (74.2)	35 (29.2)
	
	1,000,000	111 (92.5)	54 (45.0)
	
	Infinite	110 (91.7)	60 (50.0)

4.0	200,000	93 (77.5)	44 (36.7)
	
	1,000,000	116 (96.7)	63 (52.5)
	
	Infinite	114 (95.0)	65 (54.2)

4.5	200,000	97 (80.8)	48 (40.0)
	
	1,000,000	117 (97.5)	72 (60.0)
	
	Infinite	115 (95.8)	76 (63.3)

5.0	200,000	102 (85.0)	57 (47.5)
	
	1,000,000	117 (97.5)	79 (65.8)
	
	Infinite	115 (95.8)	90 (75.0)

The row with "*C*" = 2 and "Population size" = 200,000 identifies the numbers and percentages of confidence intervals containing their targets of mortality risks at selected birthweights (three for each of four mixture components), based on 10 repetitions in each of which 25 samples of size 50,000 were simulated from a 4-component normal mixture. Results under the heading of "Bias adjustment included" are based on Equation (6) with *C *= 2. Results under the heading of "Bias adjustment omitted" are based on Equation (5) with *C *= 2. The 25 samples of size 50,000 had overlap consistent with a population size of 200,000. Other rows correspond to different choices of *C *and/or population sizes.

#### b. Another example with real data

**Figure 2:**
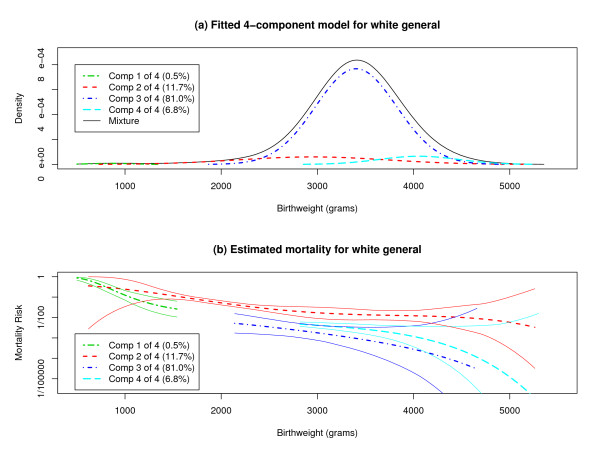
**Mixture Modeling Results and Mortality for White Singleton Infants**. (a) A 4-component normal mixture model, with parameters estimated by combining the results for 25 samples of size 50,000 from the population of white singletons in general, is shown. (b) Estimated birthweight specific-mortality curves are presented for each component of a 4-component normal mixture model, along with confidence bounds determined by Equations (6) and (7) with *C*_0 _= 4.0 and *φ *= .0055 based on 25 samples of size 50,000 from the population of white singletons in general.

We drew *N*_*rep *_= 25 samples of size 50,000 from the population of 9,162,303 white singletons born from 2000 to 2002, without regard to maternal smoking status. Figure 2a and Table 4 present overall estimates and confidence intervals for parameters in a 4-component normal mixture, while Figure 2b and Table 5 pertain to mortality risks. Confidence intervals for mixture parameters are based on Equations (7) and (8) in the previous paper with *φ *= .0055 = 50,000/9,162,303 and *C*_0 _= 2.5. Confidence intervals for mortality risks are based on Equations (6) and (7) in the present paper with *φ *= .0055 and *C*_0 _= 4.0.

**Table 4:** Mixture Modeling Results for White Singleton Infants

Quantity	**p**_**1**_	**p**_**2**_	**p**_**3**_	**p**_**4**_
$\overset{\wedge }{\theta }$ [average of 25 estimates]	.005	.117	.810	.068

${\overset{\wedge }{S}}_{\theta }$ [standard deviation of 25 estimates]	.001	.010	.012	.006

${\overset{\wedge }{B}}_{\theta }$ [bias adjustment]	.001	.012	.011	.007

Confidence interval	(.004, .006)	(.099, .135)	(.792, .827)	(.058, .078)

Quantity	μ_1_	μ_2_	μ_3_	μ_4_

$\overset{\wedge }{\theta }$ [average of 25 estimates]	862	2948	3402	4056

${\overset{\wedge }{S}}_{\theta }$ [standard deviation of 25 estimates]	60	52	6	18

${\overset{\wedge }{B}}_{\theta }$ [bias adjustment]	22	47	4	36

Confidence interval	(809, 915)	(2874, 3021)	(3395, 3410)	(4011, 4100)

Quantity	σ_1_	σ_2_	σ_3_	σ_4_

$\overset{\wedge }{\theta }$ [average of 25 estimates]	233	776	421	416

${\overset{\wedge }{S}}_{\theta }$ [standard deviation of 25 estimates]	40	23	5	19

${\overset{\wedge }{B}}_{\theta }$ [bias adjustment]	42	25	5	11

Confidence interval	(170, 295)	(739, 813)	(413, 429)	(395, 437)

Parameters in a 4-component normal mixture model for birthweight distribution are estimated, based on 25 samples of size 50,000 from the population of white singletons in general. Confidence intervals are constructed using Equations (6) and (7) with *C*_0 _= 2.5 and *φ* = .0055.

**Table 5:** Mortality for White Singleton Infants

Quantity	@ 1000 g	@ 2000 g	@ 3000 g	@ 4000 g
Risk in component 1: logit^-1^{$\overset{\wedge }{\theta }$} [point estimate] Confidence interval	124.3 (71.1, 208.4)	---	---	---

Risk in component 2: logit^-1^{$\overset{\wedge }{\theta }$} [point estimate] Confidence interval	242.8 (34.3, 743.1)	52.1 (41.3, 65.6)	17.0 (9.0, 31.9)	12.1 (6.6, 22.2)

Risk in component 3: logit^-1^{$\overset{\wedge }{\theta }$} [point estimate] Confidence interval	---	---	1.8 (0.8, 4.1)	0.3 (0.02, 3.9)

Risk in component 4: logit^-1^{$\overset{\wedge }{\theta }$} [point estimate] Confidence interval	---	---	4.2 (3.1, 5.7)	1.2 (0.4, 3.8)

Odds ratio, component 1 vs. component 2: exp{$\overset{\wedge }{\theta }$} [point estimate] Confidence interval	0.44 (0.03, 6.90)	---	---	---

Odds ratio, component 2 vs. component 3: exp{$\overset{\wedge }{\theta }$} [point estimate] Confidence interval	---	---	9.77 (2.35, 40.6)	44.3 (2.55, 768)

Odds ratio, component 2 vs. component 4: exp{$\overset{\wedge }{\theta }$} [point estimate] Confidence interval	---	---	4.15 (2.04, 8.43)	10.4 (3.24, 33.6)

Odds ratio, component 3 vs. component 4: exp{$\overset{\wedge }{\theta }$} [point estimate] Confidence interval	---	---	0.42 (0.18, 1.01)	0.24 (0.01, 5.34)

Mortality risks and odds ratios are estimated at selected birthweights, based on 25 samples of size 50,000 from the population of white singletons in general. Confidence intervals are constructed using Equations (6) and (7) with *C*_0 _= 4.0 and *φ *= .0055.

The confidence intervals for *p*_2_, *p*_3_, *μ*_2_, *σ*_4 _are considerably narrower than they were for white singletons born to heavily-smoking mothers, as are the confidence bounds for *r*_1_(*x*) in the VLBW range, *r*_2_(*x*) in the MLBW range, and *r*_3_(*x*) in much of the NBW range. The confidence bounds for *r*_2_(*x*) do not overlap those for *r*_3_(*x*) or *r*_4_(*x*) anywhere in the NBW range, indicating heterogeneity in mortality risk. In particular, the odds of mortality at 3000 g are an estimated 9.77 times as large in component 2 as in component 3 (95% confidence interval, 2.35 to 40.6) and an estimated 4.15 times as large in component 2 as in component 4 (95% confidence interval, 2.04 to 8.43).

The reason that mixture parameters and mortality risks are estimated more precisely for white singletons in general than for white singletons born to heavily-smoking mothers is that *N*_*rep *_= 25 samples of size 50,000 from a population of 9,162,303 contain approximately 1,171,467 distinct records, far more than the approximately 202,677 distinct records contained in *N*_*rep *_= 25 samples of size 50,000 from a population of 202,849. (Section II of [Additional file 1] from our previous paper provides a formula from which one may approximate the number of distinct records in multiple samples from the same population.) Even more precise estimation is possible for white singletons in general if *N*_*rep *_is taken larger.

## Discussion

This paper completes a two-part series on a new framework for modeling birthweight distributions and fetal-infant mortality. The main advantage of this new framework is its potential to reveal heterogeneity in mortality risk that may be undetectable if one relies on a contaminated normal model or 2-component normal mixture to represent a birthweight distribution.

With the contaminated normal model, the lower residual distribution and the predominant distribution have little overlap. As such, there is little overlap in the ranges of birthweights over which each component has a well-defined risk function. This is depicted in Figure 1b, where the red and green dashed curves do not occupy the same birthweights except for a small interval near 1700 g. Thus, except for birthweights close to 1700 g, the contaminated normal model effectively imposes a unique mortality risk for all infants at any fixed birthweight. This occurs because the contaminated normal model classifies all NBW cases, along with almost all MLBW and HBW cases, as originating from the predominant distribution, while it classifies virtually all VLBW and ELBW births as arising from the lower residual distribution. Yet, presumably some compromised pregnancies yield MLBW, NBW, and HBW births. Hence, not only does the estimated proportion .975 overstate the fraction of uncompromised pregnancies, but also no distinction can be made between compromised and uncompromised pregnancies at birthweights above 1700 g.

In contrast, the 2-component normal mixture has some ability to reveal heterogeneity. However, this ability is limited to the MLBW, NBW, and HBW ranges. As shown in Figure 1c, the 2-component normal mixture effectively imposes a unique mortality risk at each birthweight in the VLBW and ELBW ranges. At first glance, that may not seem worrisome. After all, the MLBW, NBW, and HBW cases may arise from a mix of compromised and uncompromised pregnancies, while presumably the VLBW and ELBW cases arise almost exclusively from compromised pregnancies. Yet, implicit in the 2-component normal mixture is a belief that all compromised pregnancies are qualitatively similar, in the sense of sharing a common birthweight-specific mortality curve. Perhaps such a belief is approximately valid for some populations. Unfortunately, the 2-component normal mixture imposes this belief mathematically and does not provide any way for it to be tested empirically. The framework that we have presented, on the other hand, allows such a belief to be tested empirically. Indeed, the example in Section 3b of Results shows that component 2 in the population of white singletons has demonstrably higher mortality risk at some birthweights than component 4 in the same population. We regard component 3 as most plausibly representing uncompromised pregnancies in this population, so that components 2 and 4 most plausibly consist of compromised pregnancies. Therefore, not all compromised pregnancies in this population share a common birthweight-specific mortality curve.

The components identified in our empirical explorations are undoubtedly related to gestational age. While detailed speculations about the precise nature of the relationship are premature, one or more of the components may have an elevated rate of intrauterine growth restriction (IUGR). Typically, IUGR is measured in population-based vital statistics data as births below (variously) the 5th or 10th percentile of birthweight for gestational age. Other aspects not presently measured on birth certificates in the United States include head circumference at birth, birth length (i.e., crown-heel length or crown-rump length), and waist/hip ratio. However IUGR might be quantified, its frequency within each component could be estimated as indicated in the next paragraph.

A useful extension of our methodology would entail probabilistically relating a covariate of interest, such as gestational age or IUGR, to the mixture components. Suppose that the covariate of interest were dichotomous. For gestational age, we could create a dichotomy by labeling infants as "preterm" or "term". Then, given a fitted *k*-component mixture model for birthweight distribution, we could apply PMLR with dichotomized gestational age or IUGR rather than mortality as the dependent variable. The resulting ${\overset{\wedge }{r}}_{j}(x)$ would denote not the estimated mortality risk but rather the estimated probability of a preterm birth or of IUGR as a function of birthweight within component *j *(1 <= *j *<= *k*). To estimate the overall probability of a preterm birth or of IUGR within component *j*, we would integrate ${\overset{\wedge }{r}}_{j}(x)$ over the estimated distribution of birthweights within component *j*,

(10) $$\int {\overset{\wedge }{r}}_{j}(x)f(x;{\overset{\wedge }{\mu }}_{j},{\overset{\wedge }{\sigma }}_{j})dx.$$

Pursuing this idea and extending it to multiple covariates, both categorical and continuous, would enable us to describe the joint distribution of covariates within each mixture component. If the joint distributions of covariates within different mixture components had little overlap, then we would be able to assert an approximate correspondence between the mixture components and identifiable subpopulations with biological meaning. Such discoveries would provide greater epidemiologic insight into the relationships among fetal-infant mortality and its prognostic factors.

## Conclusions

The present paper, the second in a two-part series, develops a new and flexible approach to modeling fetal-infant mortality through the estimation of separate birthweight-specific mortality curves within each component of a normal mixture model describing a birthweight distribution, the number of components having been determined from the data rather than fixed *a priori*. This approach allows the detection of heterogeneity in mortality that cannot be found with a contaminated normal model or a 2-component normal mixture model. A 2-component normal mixture model assumes that infants from compromised pregnancies share a common birthweight-specific mortality curve, while a contaminated normal model assumes that all infants share a common curve over some (possibly quite large) interval of birthweights. Yet, our approach has demonstrated that components 2 and 4 in a 4-component normal mixture model for white singleton birthweights have distinct birthweight-specific mortality curves. Since components 2 and 4 in this population most plausibly consist of compromised pregnancies, we see that infants from compromised pregnancies need not share a common birthweight-specific mortality curve. Finally, this paper lays some groundwork for future research aimed at discovering approximate correspondences between mixture model components and identifiable subpopulations.

## Methods

[Additional file 1] presents technical details on our methodology and its implementation.

## Abbreviations

ELBW: extremely low birthweight; FLIC: Flexible Information Criterion; HBW: high birthweight; IUGR: intrauterine growth restriction; MLBW: moderately low birthweight; NBW: normal birthweight; NCHS: National Center for Health Statistics; PMLR: parametric mixtures of logistic regressions; VLBW: very low birthweight

## Competing interests

The authors declare that they have no competing interests.

## Authors' contributions

RC - Concept and design, analysis and interpretation of data, drafting of the manuscript, critical revision of the manuscript for important intellectual content, statistical analysis, read and approved final manuscript. LWC - Concept and design, acquisition of data, analysis and interpretation of data, drafting of the manuscript, critical revision of the manuscript for important intellectual content, read and approved final manuscript. TL - Analysis and interpretation of data, drafting of the manuscript, critical revision of the manuscript for important intellectual content, read and approved final manuscript. RSK - Analysis and interpretation of data, drafting of the manuscript, critical revision of the manuscript for important intellectual content, read and approved final manuscript.

## Pre-publication history

The pre-publication history for this paper can be accessed here:

http://www.biomedcentral.com/1471-2393/10/44/prepub

## Supplementary Material

Additional file 1 **Technical Appendix**. Additional file 1 presents technical details on our methodology and its implementation. Click here for file
