# Supplementary material for: Thinking outside the curve, part II: modeling fetal-infant mortality
Source: BMC Pregnancy Childbirth. 2010 Aug 12;10:44. doi: 10.1186/1471-2393-10-44 (PMC2936298; doi:10.1186/1471-2393-10-44)
Supplement: Additional file 1 — Technical Appendix. Additional file 1 presents technical details on our methodology and its implementation. [file 1471-2393-10-44-S1.DOC]

**Additional File 1 (Technical Appendix) for**

Thinking outside the curve, part II:

modeling fetal-infant mortality

Richard Charnigo,^1*^ Lorie W. Chesnut,^2^ Tony LoBianco,^3^ Russell S. Kirby^4^

^1^Departments of Statistics and Biostatistics

University of Kentucky

Lexington, KY 40506-0027, USA

^2^Department of Epidemiology

University of Kentucky

Lexington, KY 40536-0003, USA

^3^Interdisciplinary Human Development Institute

University of Kentucky

Lexington, KY 40506-0051, USA

^4^Department of Community and Family Health

University of South Florida

Tampa, FL 33612, USA

* corresponding author

RC RJCharn2@aol.com

LWC lorie.chesnut@uky.edu

TB tflobi1@email.uky.edu

RSK rkirby@health.usf.edu

**I. Computational issues in mortality risk estimation**

Our implementation of parametric mixtures of logistic regressions (PMLR) for estimating birthweight-specific mortality curves [7] relies on the optimization (optim) procedure in version 2.3.1 of R. We apply optim to maximize the likelihood of observed mortality outcomes conditional on the estimated mixture parameters. This likelihood is expressed in terms of 5*k* mortality parameters, where *k* is the number of mixture components. The 5 mortality parameters for each mixture component are the 5 coefficients in the polynomial representation of *p_j_*(*x*) from Equation (2), namely , where *z* = (*x* – 3000)/1000 and *x* is birthweight in grams.

For initial values we take = -5 and for *j* between 1 and *k*. These initial values are consistent with a population mortality rate of 6.7 per 1000 and do not impose any *a priori* beliefs of heterogeneity in mortality risk across or within components. However, we do impose the following constraints: -9 <= <= -1, -6 <= <= 6, -3 <= <= 3, -1.5 <= <= 1.5, and -0.75 <= <= 0.75.

Three practical considerations for assessing the adequacy of the optim-based estimates are as follows.

First, estimates exactly equal to constraints (e.g., = 0.75) are suspect. At best, the global optimum is on the boundary of the parameter space and the researcher should reconsider the constraints; at worst, optim has failed to detect even a local optimum.

Second, if a researcher applies PMLR to *N_rep_* data sets, some anomalies may be apparent upon juxtaposition of mortality parameter estimates from the different data sets. For instance, an estimate of 5.05 for followed by estimates of -1.02, -0.70, -0.84, and -0.93 for from four other data sets suggests a problem with the 5.05.

Third, plotting logit ^-1^[] as a function of *x* (recall, *z* is a linear rescaling of *x*) may indicate whether the estimates of , , , , and are collectively reasonable. A caveat is not to overreact if extremely small or large *x* yield strange values of logit ^-1^[], unless extremely small or large *x* arise frequently within component *j*. For instance, estimated mortality at 1000g is essentially meaningless for components 3 and 4 in a 4-component model.

**II. Rationale for the logit transformation in mortality risk estimation**

To obtain a confidence interval for *r_j_*(*x*_0_), where 1 <= *j* <= *k* and *x*_0_ is a fixed birthweight, we actually construct a confidence interval for logit{ *r_j_*(*x*_0_) } and then apply the inverse logit transformation to the endpoints of this confidence interval. That is, we take = logit{ *r_j_*(*x*_0_) } in Equation (6) instead of setting = *r_j_*(*x*_0_). There are two reasons for doing this.

First, setting = *r_j_*(*x*_0_) would imply an overall estimate of the form

, which is not in general the inverse logit of a degree-four polynomial in *z =* (*x* – 3000)/1000. Taking = logit{ *r_j_*(*x*_0_) } allows us to use the overall estimate from Equation (4), which is the inverse logit of a degree-four polynomial in *z* because, with obvious notation,

= logit ^-1^[ {} ]

and a sum of *N_rep_* degree-four polynomials is another degree-four polynomial. Thus, the overall estimate of the risk function for component *j* has the same mathematical form as the estimate based on a single sample.

Second, and more importantly, the logit transformation guarantees that the confidence interval for *r_j_*(*x*_0_) will have lower endpoint greater than 0 and upper endpoint less than 1 without any ad-hoc truncations.
